# Characteristics of Heavy Metals and Pb Isotopic Composition in Sediments Collected from the Tributaries in Three Gorges Reservoir, China

**DOI:** 10.1155/2014/685834

**Published:** 2014-01-28

**Authors:** Bo Gao, Huaidong Zhou, Yong Huang, Yuchun Wang, Jijun Gao, Xiaobo Liu

**Affiliations:** ^1^Department of Water Environment, China Institute of Water Resources and Hydropower Research, Beijing 100038, China; ^2^State Key Laboratory of Simulation and Regulation of Water Cycle in River Basin, China Institute of Water Resources and Hydropower Research, Beijing 100038, China; ^3^College of Horticulture, Jilin Agricultural University, Changchun 130118, China

## Abstract

The concentrations, distribution, accumulation, and potential ecological risk of heavy metals (Cr, Cu, Zn, Ni, As, Pb, Cd, and Hg) in sediments from the Three Gorges Reservoir (TGR) tributaries were determined and studied. Pb isotopic compositions in sediments were also measured to effectively identify the potential Pb sources. The results showed that the average concentrations of heavy metals in sediment of TGR tributaries were higher than the local background values of soils and sediments in China. The assessment by Geoaccumulation Index indicated that Cu, Ni, and Hg were at the “slightly polluted” level and Cd was ranked as the “moderately polluted” level in tributary sediments of TGR. The assessment by Potential Ecological Risk Index showed that Hg and Cd were the predominant elements in tributary sediments in TGR. The Pb isotopic ratios in sediments varied from 1.171 to 1.202 for ^206^Pb/^207^Pb and from 2.459 to 2.482 for ^208^Pb/^207^Pb in TGR. All Pb isotopic ratios in sediments were similar to those from coal combustion, lead ores (the mining activities and smelting process), and cement material, indicating that these anthropogenic inputs may be the main sources for Pb pollution in sediments of TGR tributaries.

## 1. Introduction

In recent years, a large number of heavy metals were discharged into the nature environment because of anthropogenic activity such as ore mining, metal smelting, fossil oil burning, and machinery wearing. This can give rise to the accumulation of heavy metals in various environment media [1, 2]. Sediment, as an important part in the water environment, is more and more important to study heavy metals pollution in rivers because it was often regarded as major indicator to assess the heavy metal pollution in water environment. In fact, heavy metals in sediments can be released into water when water chemical and hydrological condition changed [3]. Therefore, heavy metals in sediments can pose a threat to the water quality safety and aquatic organisms [4, 5].

The Three Gorges Dam, in China, is the world's largest dam with 2335 m length and 185 m height. With the completion of the Three Gorges Dam (2003), the Three Gorges Reservoir (TGR) became the biggest reservoir in China, creating a total area of 1080 km^2^ in 2009. The water level of the reservoir fluctuation resulted in the formation of the water-level-fluctuation zone with a total area of 350 km^2^ in the reservoir. The TGR plays important role in economic development and national drinking water safety.

However, how a dam impacts the local environment in TGR is still unknown. There is also little information available about the characteristic of heavy metals in the sediments in this huge reservoir, especially for the period of its submergence. Increased shipping and industrial waste will influence the deposition of heavy metals which have been accumulating in the water-level-fluctuation during submergence period. In the downstream of the reservoir, intensive land use has also increased nonpoint pollutants in the reservoir region. The primary objectives of the present study were (1) to provide basic information of the concentration and distribution of the heavy metal contamination in sediments of TGR tributaries after the submergence, (2) to perform sediment pollution assessment using the Geoaccumulation Index (*I*_geo_) and the Potential Ecological Index, and (3) to trace the sources of Pb pollution in sediments using Pb isotopic ratios. This study provides relevant information of the heavy metal contamination in tributary sediments of TGR after the submergence. These data will form the basis for the comparison with future data related to heavy metal pollution in sediments and be useful for the development of management decisions, pollution-control, and sediment remediation strategies in this region.

## 2. Materials and Methods

### 2.1. Sample Sites

**Figure 1:**
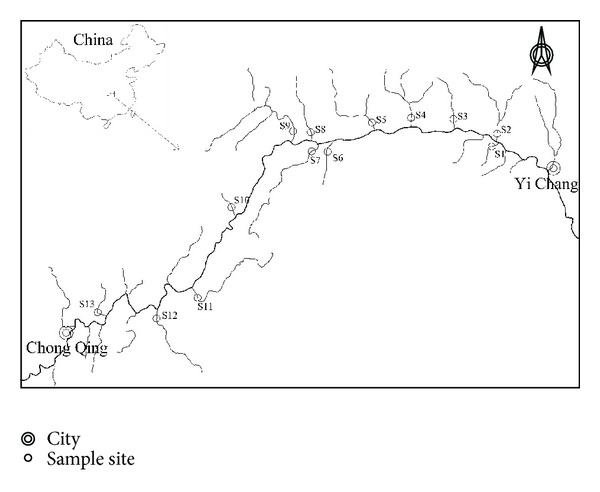
Map of the study area and sampling sites in TGR.

The tributary sediments were collected from the TGR, China. A map of the sampling locations is shown in Figure 1. The TGR area is located in China, west of Hubei and east of Chongqing city (28°32′–31°44′N and 105°44′–111°39′E), covering an overall area of 58,000 km^2^ and including totally 20 districts and counties (cities). TGR is the largest hydroelectric project ever built in China, as well as in the world. After the Three Gorges Dam was constructed at Sandouping, a large dam formed in the upstream direction of the Yangtze River, with the length of over 600 km. The reservoir waters and their fringe areas are generally called the TGR area of the Yangtze River.

### 2.2. Sample Collection

Twenty-six surface sediments (two samples for each tributary) were collected from thirteen major tributaries of TGR in March 2009 after its submergence period. The sampling tributaries were described within the zone (Figure 1). At each sampling site, sediment samples were taken using a stainless steel collector near the middle of the flow of the stream. About 1 kg of sediments was collected into clean polyethylene bags and treated immediately on returning to the laboratory. The sediment samples were wetsieved through an acid-cleaned 63 *μ*m mesh nylon sieve in order to obtain the chemically active material, dried at −40°C to constant weight, and ground in an agate mortar to ensure homogeneity.

### 2.3. Analytical Methods

All chemical treatments were in the ultraclean laboratory, and all reagents were of high purity grade. Total metal concentrations in the sediments were measured using the established method [6]. Briefly, a mass of 40 mg of dry sample was weighed and dissolved into 10 mL Teflon bombs. About 2 mL concentrated HNO_3_ + 0.2 mL concentrated H_2_O_2_ were added to samples and left on a hot plate for one day. This step was to remove organic materials from sediment samples. The samples were then dried at 120°C. The residue was dissolved in 1 mL HNO_3_ + 1 mL HF of samples. After 30 min ultrasonic procedure, the samples were taken into sealed bombs and were placed in an oven at 190°C for 48 h. This procedure resulted in clear solutions for sediment sample. After evaporation at 120°C, samples were dissolved in 1% HNO_3_. Inductively coupled plasma-mass spectrometry (ICP-MS, Perkin Elmer Elan DRC-e) was used to determine the total concentration of Cd, Cr, Cu, Ni, Pb, As, and Zn. Mercury in sediments was determined by the Direct Mercury Analyzer (Milestone DMA-80). The accuracy of the analytical procedures employed for the analysis of the metals in sediments was checked using the certified China reference material of stream sediment (GSD-12, GBW07312), obtaining good agreement with certified values.

### 2.4. Pb Isotopic Measurement

Pb isotopic analyses were separated using microexchange columns of anion resin of Dowex-I (200–400 mesh) and HBr and HCl as eluants [7]. Measurements of Pb isotopic compositions were carried out using a VG-354 mass spectrometer. The average measured values of the standard NIST SRM-981 are ^206^Pb/^204^Pb = 16.934  ±  0.007, ^207^Pb/^204^Pb = 15.486  ±  0.012, and ^208^Pb/^204^Pb = 36.673  ±  0.033 (*n* = 20), respectively, which are in good agreement with the recalibrated values of 16.9322, 15.4855, and 36.6856, respectively [8]. Analytical uncertainties in 2s (2s, 2 standard deviation) for Pb isotopic ratios were better than 0.1%.

### 2.5. Assessment Methods

The Geoaccumulation Index (*I*_geo_) introduced by Müller (1979) was used to assess metal pollution in sediments [9]. Geoaccumulation Index is expressed as follows:

(1) $$\begin{array}{c} I_{\text{geo}}=\text{lo}{\text{g}}_{2}\left(\frac{C_{n}}{1.5B_{n}}\right), \end{array}$$

where *C*_*n*_ is the measured concentration of heavy metal (*n*) in the sediment, *B*_*n*_ is the geochemical background value of heavy metal (*n*), and 1.5 is the background matrix correction factor due to lithogenic effects. In the present study, *B*_*n*_ was selected from the literature [10]. Geoaccumulation Index includes seven grades from Class 0 (*I*_geo_ ≤ 0) to Class 6 (*I*_geo_ ≥ 5) in Table 1.

The Potential Ecological Risk Index introduced by Häkanson was also used to assess potential ecological risk of heavy metals in sediments [11]. The Potential Ecological Risk Index (RI and *E*_*i*_) is expressed as follows:

(2) $$\begin{array}{c} E_{i}=T_{i}\left(\frac{C_{i}}{C_{i}^{n}}\right),\\ \text{RI}=\sum E_{i}=\sum T_{i}\left(\frac{C_{i}}{C_{i}^{n}}\right), \end{array}$$

where *C*_*i*_ is the measured concentration of heavy metal (*i*), *C*_*i*_^*n*^ is the geochemical background value of heavy metal (*i*), and *T*_*i*_ is the toxic coefficient of heavy metal (*i*). In this study, the toxic coefficients of Cr, Cu, Zn, Ni, As, Pb, Cd, and Hg are 2, 5, 1, 5, 10, 5, 30, and 40, respectively [12]. The potential ecological risk degree is shown in Table 2.

## 3. Results and Discussion

### 3.1. Concentrations of Heavy Metals in Sediments

The results of heavy metal concentrations in sediments of TGR tributaries are shown in Table 3. The mean concentrations of Cr, Cu, Zn, Ni, As, Pb, Cd, and Hg in sediments in TGR were 79.73, 46.67, 114.79, 41.67, 12.26, 38.11, 0.71, and 0.13 mg/kg, respectively. The mean of the heavy metals in sediments from TGR was significantly higher than the background values of soils and sediment [10, 13]. This indicated that the sediments in TGR may be contaminated by heavy metals from anthropogenic sources. In fact, the minimum concentrations of the metals including Cr, Cu, Ni, and Cd were also higher than the soils background values in China. The order of heavy metals in sediments of TGR tributaries was Zn > Cr > Cu > Ni > Pb > As > Cd > Hg. Since China government does not have its own regulatory guideline for sediments, probable effects concentration (PEC) and threshold effect concentration (TEC) [16] were used to assess the ecotoxicological level of observed metal levels. The mean concentrations of Cd, Zn, and Hg in sediments of TGR tributaries were lower than TEC values, indicating that these metals were unlikely to result in harmful effects. However, sediments samples with the mean concentration of other metals between PEC and TEC may be toxic for aquatic organism. Heavy metals concentrations in sediments of other Chinese rivers were also presented in Table 3. It can be seen that the concentrations of heavy metals in sediments of TGR tributaries were generally lower than the downstream in Yangtze River. This result may be attributed to the differences of geographical characteristic, industrial development in the downstream of Yangtze River, especially for the high-speed development of large cities. In fact, several Chinese larger cities are located in the downstream of Yangtze River, such as Wuhan, Shanghai, and Nanjing. In addition, since the TGR was in the upstream of Yangtze River, heavy metals in sediments or suspended particles can be transferred from the upstream to downstream with the water flowing in Yangtze River.

### 3.2. Spatial Distribution of Heavy Metals in Sediments

**Figure 2:**
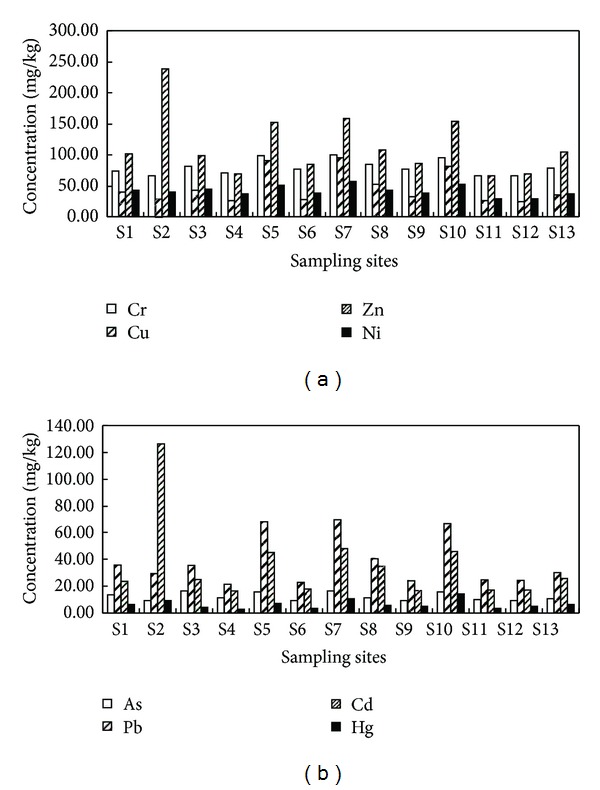
Spatial distribution of heavy metals in sediments from TGR tributaries.

From Figure 2 (Cd and Hg concentrations were 50 times the actual concentrations to better comparison with other metals), it can be seen that the mean concentrations of Cr, Cu, Ni, As, Pb, and Hg in midstream (including from S5 to S10) of TGR were generally higher than those in downstream (including from S1 to S4), and the lowest value of these metals was located in upstream (including from S11 to S13), as in Figure 1. But the order of the mean concentrations of Zn and Cd was downstream > midstream > upstream. This indicated that the contaminations of Zn and Cd were more seriously in upstream. The heavy metals in sediments from TGR tributaries showed a similar spatial distribution, except for Zn and Cd. Among tributaries of TGR, the highest value of Zn and Cd were observed at site S2 (XiangXi River). In addition, the highest concentrations of Cr, Cu, Ni, As, and Pb were found in S7 (the tributary of Modao River), and the highest concentration of Hg was observed at S10 (Ruxi River).

### 3.3. The Correlation among Heavy Metals in Sediments

To explore the geochemical behaviors of heavy metals in surface sediments from TGR tributaries, the correlations among heavy metals in sediments are presented in Table 4. In fact, Hg showed an obvious correlation with other elements in sediments, suggesting that Hg possibly existed at various pollutants. There were significant correlation coefficients among Cr, Cu, Zn, As, and Cd in sediments, suggesting that they may have similar pollution sources. In addition, Pb showed only obvious correlations with Ni, demonstrating that these two metals have common sources in sediments. According to the previous studies of heavy metals, the pollution sources of heavy metals may be complicated from several sources [17, 18]. In this present study, the main pollution sources may be industrial pollution especially for smelting metal and fuel combustion.

### 3.4. Pollution Assessment

The results of the *I*_geo_ values and pollution level of heavy metals of sediments in TGR tributaries were shown in Table 5. In the sediments of TGR tributaries, the average *I*_geo_ values of Cr, Cu, Zn, Ni, As, Pb, Cd, and Hg are −0.21, 0.29, −0.06, 0.02, −0.49, −0.17, 1.99, and 0.27, respectively. Distinctly, the average *I*_geo_ value of Cd was significantly higher than other metals in the sediments, which was ranked as “moderately polluted” level. The highest contamination level of Cd was observed in sampling sites of S2, which was ranked as “strongly to extremely polluted” level. In addition, the average *I*_geo_ of Cu, Ni, and Hg was lower than 1, which was ranked as “slightly polluted” level. In detail, the *I*_geo_ values of Cu and Hg in sampling sites such as S5, S7, and S10 were more than 1, which was ranked as “moderately polluted” level. Next, the average *I*_geo_ values of Cr, Zn, As, and Pb were lower than 0, which were ranked as “unpolluted” level. But the *I*_geo_ values of Cr, Zn, and Pb in sampling sites such as S2, S5, S7, and S10 were more than 0, which were ranked as “slightly polluted” or “moderately polluted” level.

The results of the potential ecological risk level of heavy metals in sediment of TGR tributaries were shown in Table 6. Among the average *E*_*i*_ values of Cr, Cu, Zn, Ni, As, and Pb in sediments were 2.61, 10.32, 1.55, 7.74, 10.95, and 7.33, respectively. The average *E*_*i*_ values of these metals in all sampling sites were lower than 40, which were ranked as slightly ecological risk level. Moreover, the average *E*_*i*_ value (80.18) of Hg was more than 80 and lower than 160, which was ranked as strongly ecological risk level. In fact, Hg pollution in S10 was reached at very strongly ecological risk level. The average *E*_*i*_ value (219.06) of Cd was more than 160 and lower than 320, which was reached at very strongly ecological risk level.

The average RI value of the heavy metals in sediments from TGR tributaries was 347.50, which was between 300 and 600. This indicated that the heavy metals in sediments collected from TGR tributaries exhibited strongly potential ecological risk level, especially for Cd and Hg. The order of the potential ecological risk of heavy metals in surface sediment from TGR tributaries was Cd > Hg > As > Cu > Ni > Pb > Cr > Zn, which was corresponding to not only concentration level but also ecology toxic coefficient for heavy metals.

### 3.5. Lead Isotopic Signatures

**Figure 3:**
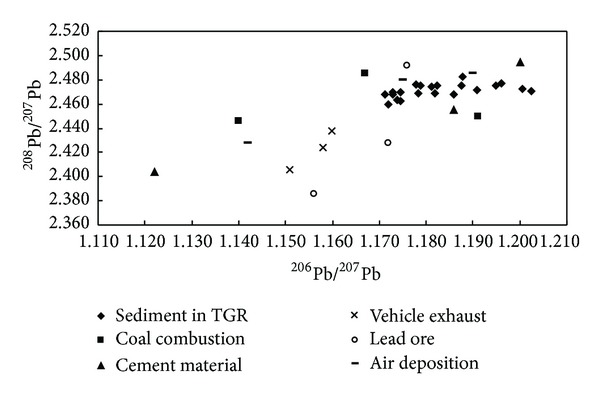
The distribution of Pb isotopic compositions in sediments from TGR and other environmental sources.

In order to identify potential Pb sources of sediment, the Pb isotopic compositions of sediment samples were analyzed. The results of Pb isotope ratios of sediment samples and other environmental sources [8, 19–21] were shown in Figure 3. Lead isotopic ratios in sediments from TGR tributaries ranged from 1.171 to 1.202 for ^206^Pb/^207^Pb and 2.459 to 2.482 for ^208^Pb/^207^Pb, which varied with other different environmental media. The average values of Pb isotope ratios (^206^Pb/^207^Pb and ^208^Pb/^207^Pb) were 1.183 and 2.471. In fact, geochemical background Pb generally has relatively high ^206^Pb/^207^Pb (~1.200), while low ^206^Pb/^207^Pb ratios (<1.190) may indicate potential anthropogenic inputs. Thus, it can be seen that ratios of ^206^Pb/^207^Pb in most sediments from TGR were lower than 1.200, suggesting the influence of anthropogenic inputs on TGR. In order to assess Pb contamination and identify potential Pb sources of sediments, the correlation ^206^Pb/^207^Pb and ^208^Pb/^207^Pb ratios in sediments were also analyzed. The results showed that the relationship was not obviously correlated (*R* ^2^ = 0.2863), indicating that Pb sources of sediments were relatively complicated and cannot be simply attributed to binary mixing process of two sources [22].

The ^206^Pb/^207^Pb and ^208^Pb/^207^Pb ratios in sediments were significantly higher than those from emission from vehicle exhaust (Figure 3). This indicated that vehicle exhaust may not be the main source responsible for Pb pollution in this region. Another research has also confirmed that lead concentrations of atmospheric aerosols in China were decreased significantly after the leaded gasoline ban [23]. However, all Pb isotopic ratios in sediments were similar to those from coal combustion and cement material, indicating that these two anthropogenic sources may be the main sources for Pb pollution in the sediments of TGR tributaries. In fact, coal was still the important source of energy for economic and industrial development in cities around TGR. With the rapid development of large cites (Chongqing) in upstream of TGR, the building construction of sites may be producing a large number of waste materials made by cement material. In addition, the ^206^Pb/^207^Pb and ^208^Pb/^207^Pb ratios in sediments were similar to lead ores and air deposition, suggesting that these sources may be the other major sources for Pb pollution in the TGR. In fact, as an important transportation of heavy metals, air deposition contained the suspended particles with high Pb concentrations [24, 25]. These air particles can enter into water environment and then deposit into the sediments.

## 4. Conclusion

Our investigation showed that the mean concentrations of Cr, Cu, Zn, Ni, As, Pb, Cd, and Hg were obviously higher than the background values of sediments and soils in China. The results of spatial distribution of heavy metals in sediments showed that heavy metals in sediments from TGR tributaries showed a similar spatial distribution, except for Zn and Cd. Among the eight metals, Cr, Cu, Zn, As, and Cd showed remarkable correlation with each other. However, Pb showed good correlation with Ni. The assessment by Geoaccumulation Index indicated that Cr, Zn, As, and Pb were ranked as the unpolluted level, while Cu, Ni, and Hg were classified as the slightly polluted level. Cd was at moderately polluted level. The assessment by Potential Ecological Risk Index indicated that Cr, Cu, Zn, Ni, As, and Pb were viewed at slightly ecological risk level. However, Hg was ranked as the strongly ecological and Cd was reached at very strongly risk level in sediments, suggesting that Hg and Cd are the predominant elements in TGR sediments. Lead isotopic ratios ranged from 1.171 to 1.202 for ^206^Pb/^207^Pb and 2.459 to 2.482 for ^208^Pb/^207^Pb in sediments from TGR tributaries and Pb sources in sediments were complicated (more than two sources) to identify. All Pb isotopic radios in sediments were similar to those from coal combustion, lead ores (the mining activities and smelting), and cement material, indicating that these anthropogenic sources may be the main sources for Pb pollution in the sediments of TGR tributaries.

## Figures and Tables

**Table 1:** Geoaccumulation Index and classification of pollution degree.

*I*_geo_ value	Classification	Pollution level
<0	0	Unpolluted
0~1	1	Slightly polluted
1~2	2	Moderately polluted
2~3	3	Moderately to strongly polluted
3~4	4	Strongly polluted
4~5	5	Strongly to extremely polluted
>5	6	Extremely polluted

**Table 2:** Description of potential ecological risk degree.

*E*_*i*_	Ecological risk of single metal	RI	Ecological risk of all metals
*E*_*i*_ < 40	Slightly	RI < 150	Slightly
40 ≤ *E*_*i*_ < 80	Moderately	150 ≤ RI < 300	Moderately
80 ≤ *E*_*i*_ < 160	Strongly	300 ≤ RI < 600	Strongly
160 ≤ *E*_*i*_ < 320	very strongly	600 ≤ RI	Very strongly
320 ≤ *E*_*i*_	Extremely		

**Table 3:** The levels of heavy metals in surface sediments from TGR tributaries and other rivers in China.

Location	Chemical element								Reference
	Cr	Cu	Zn	Ni	As	Pb	Cd	Hg	
TGR									
Max.	99.92	95.18	238.73	57.24	16.65	69.96	2.52	0.29	This study
Min.	65.93	25.07	66.92	29.72	9.14	21.21	0.33	0.06	
Mean	79.73	46.67	104.28	41.67	12.26	38.11	0.71	0.13	
Soil background values	61.0	22.6	74.2	26.9	11.2	26.0	0.097	0.065	[10]
Sediment background	82.0	35.0	78.0	33.0	9.6	27.0	0.3	0.08	[13]
Yangtze River (Wuhan)	87.82	51.64	140.27	40.91	15.85	45.18	1.53	0.15	[14]
Lower reach of Yangtze River	98.32	48.61	129.73	41.49	13.54	50.77	2.82	0.16	[15]
TEC	43.4	31.6	121.0	22.7	9.8	35.8	1.0	0.18	[16]
PEC	111.0	149.0	459.0	48.6	33.0	128.0	5.0	1.06	[16]

**Table 4:** Analysis of correlation about heavy metals in surface sediments from TGR tributaries.

Element	Cr	Cu	Zn	Ni	As	Pb	Cd	Hg
Cr	1							
Cu	0.922^a^	1						
Zn	0.960^a^	0.924^a^	1					
Ni	0.333	0.546	0.462	1				
As	0.823^a^	0.853^a^	0.846^a^	0.282	1			
Pb	0.040	0.281	0.189	0.948^a^	0.030	1		
Cd	0.926^a^	0.907^a^	0.990^b^	0.519	0.848^a^	0.256	1	
Hg	0.625^b^	0.735^a^	0.727^a^	0.737^a^	0.562^b^	0.559^b^	0.785^a^	1

^a^Correlation is significant at the 0.01 level (2-tailed).

^b^Correlation is significant at the 0.05 level (2-tailed).

**Table 5:** The Geoaccumulation Index and classification of heavy metals in sediments from TGR tributaries.

Sampling site	*I*_geo_ value/classification							
	Cr	Cu	Zn	Ni	As	Pb	Cd	Hg
S1	−0.29/0	0.25/1	−0.14/0	0.08/1	−0.31/0	−0.13/0	1.70/2	0.39/1
S2	−0.47/0	−0.23/0	1.10/2	−0.04/0	−0.87/0	−0.40/0	4.12/5	0.92/1
S3	−0.17/0	0.36/1	−0.17/0	0.13/1	−0.05/0	−0.13/0	1.77/2	−0.08/0
S4	−0.37/0	−0.34/0	−0.69/0	−0.12/0	−0.57/0	−0.88/0	1.16/2	−0.68/0
S5	0.11/1	1.41/2	0.45/1	0.34/1	−0.07/0	0.81/1	2.64/3	0.59/1
S6	−0.26/0	−0.26/0	−0.38/0	−0.06/0	−0.88/0	−0.76/0	1.29/2	−0.50/0
S7	0.13/1	1.49/2	0.51/1	0.50/1	−0.01/0	0.84/1	2.73/3	1.17/2
S8	−0.11/0	0.64/1	−0.04/0	0.11/1	−0.53/0	0.08/1	2.28/3	0.30/1
S9	−0.24/0	−0.05/0	−0.37/0	−0.06/0	−0.80/0	−0.67/0	1.22/2	0.004/1
S10	0.06/1	1.26/2	0.46/1	0.37/1	−0.06/0	0.77/1	2.66/3	1.56/2
S11	−0.47/0	−0.39/0	−0.73/0	−0.44/0	−0.75/0	−0.63/0	1.22/2	−0.51/0
S12	−0.45/0	−0.44/0	−0.68/0	−0.44/0	−0.86/0	−0.68/0	1.25/2	−0.04/0
S13	−0.22/0	0.08/1	−0.09/0	−0.11/0	−0.66/0	−0.37/0	1.83/2	0.36/1
Average	−0.21/0	0.29/1	−0.06/0	0.02/1	−0.49/0	−0.17/0	1.99/2	0.27/1

**Table 6:** The *E*_*i*_ and RI values of heavy metals in surface sediment from TGR.

Sampling sites	*E*_*i*_ value of heavy metal								RI value
	Cr	Cu	Zn	Ni	As	Pb	Cd	Hg	
S1	2.45	8.90	1.36	7.92	12.09	6.87	146.29	78.54	272.33
S2	2.16	6.40	3.22	7.31	8.23	5.67	780.00	113.67	933.98
S3	2.67	9.61	1.33	8.20	14.51	6.87	153.71	56.62	261.72
S4	2.32	5.95	0.93	6.92	10.08	4.08	100.82	37.48	175.50
S5	3.25	19.94	2.05	9.48	14.32	13.18	279.59	90.56	441.84
S6	2.50	6.28	1.15	7.19	8.16	4.43	110.10	42.37	189.37
S7	3.28	21.06	2.14	10.64	14.87	13.45	297.84	135.17	509.08
S8	2.79	11.66	1.45	8.08	10.41	7.90	218.35	74.04	342.77
S9	2.53	7.26	1.16	7.20	8.61	4.71	105.15	60.15	203.96
S10	3.12	17.96	2.06	9.72	14.42	12.82	284.85	176.32	530.97
S11	2.17	5.73	0.90	5.52	8.93	4.84	104.54	42.22	180.38
S12	2.19	5.55	0.94	5.55	8.25	4.67	107.01	58.36	198.06
S13	2.57	7.92	1.41	6.96	9.46	5.78	159.59	76.89	277.55
Average	2.61	10.32	1.55	7.74	10.95	7.33	219.06	80.18	347.50
